# MPO Expression of Background Neutrophils in MPO Negative Acute Promyelocytic Leukemia, An Easy Clue to Corroborate a Challenging Diagnosis: A Case Report and Review of Literature

**DOI:** 10.1155/2023/7979261

**Published:** 2023-12-26

**Authors:** Kritika Krishnamurthy, Jui Choudhuri, K. H. Ramesh, Yanhua Wang

**Affiliations:** ^1^Department of Pathology, Montefiore Medical Center, Bronx, NY 10467, USA; ^2^Department of Pathology, Albert Einstein College of Medicine, Bronx, NY 10467, USA

## Abstract

Acute promyelocytic leukemia (APL) is characterized by the pathogenic driver fusion transcript PML-RARA resulting from the t(15;17) translocation. Early recognition of APL with prompt ATRA induction has a decisive impact on the early death rate. The preliminary diagnosis of APL relies heavily on cytomorphology and flow cytometry. In APL with variant morphology, such as the microgranular variant, immunophenotype, especially the bright MPO positivity is the basis of diagnosis. Till date, only five cases of APL with reduced/absent MPO have been described in literature. The identification of MPO deficiency based on genetic testing would involve at the least a MPO gene scanning with NGS, followed by microarray to identify somatic uniparental disomy in heterozygotes. This testing is not only redundant given the scant clinical implications of heterozygous MPO deficiency but also time consuming. An easy way to identify background MPO deficiency confounding the immunophenotype of a myeloid neoplasm is the MPO expression in background neutrophils gated on the initial flow cytometry. A dim MPO in the background neutrophils, in the morphological setting of APL, can identify underlying MPO deficiency, clarifying the immunophenotypic ambiguity and thus establishing an unequivocal diagnosis as seen in the current case.

## 1. Introduction

Acute promyelocytic leukemia (APL) is characterized by the pathogenic driver fusion transcript PML-RARA resulting from the t(15;17) translocation [1]. Despite the revolutionary impact of all-trans retinoic acid (ATRA) therapy on the prognosis of APL, the death rate in the early phases of disease due to precipitant disseminated intravascular coagulation remains high [2, 3]. Early recognition of APL with prompt ATRA induction has a decisive impact on the early death rate [2, 3]. Thus, the preliminary diagnosis of APL relies heavily on cytomorphology and flow cytometry and is the trigger for ATRA initiation, usually before the subsequent confirmatory PML-RARA FISH or qPCR is reported [4]. Usually, the cytomorphology and-flow based diagnosis of APL is straightforward based on typical blast morphology and characteristic immunophenotype. However, variant morphology, such as paucity of Auer rods and tell-tale granules in the microgranular variant, makes it challenging in these cases with substantial reliance on immunophenotype, especially the bright MPO positivity which is discerning in these cases [5]. This report and review of literature describe a MPO negative microgranular APL, with a focus on identifying this rare presentation of microgranular variant based on conventional testing strategies.

## 2. Case Report

A 75-year-old African American woman, with history of breast cancer 17 years prior status posttreatment, colon adenocarcinoma 14 years prior status posttreatment, and currently under medical management for hypertension and chronic kidney disease, presented to the emergency room with recent onset rectal bleeding.

Her complete blood counts revealed marked leukocytosis with WBC count of 46.2 k/*μ*l, severe anemia with hemoglobin 8.4 g/dl, and severe thrombocytopenia with platelet count of 70 k/*μ*l. Her differential counts divulged a high blast percentage of 57%. Her coagulation parameters were deranged with PT of 17.6 s (INR 1.4), aPTT of 39.9 s, elevated fibrinogen of 727 mg/dl, and d-dimer >20 *μ*g/dl.

**Figure 1:**
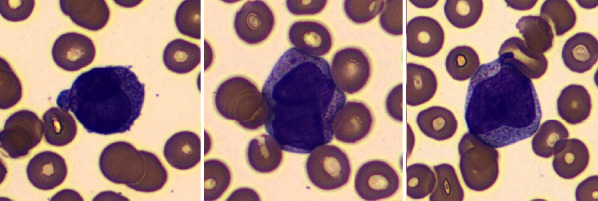
Wright-Giemsa stained peripheral blood smear showing medium to large blasts with characteristic bilobed nucleus and variable cytoplasmic granularity. Several hypogranular forms intermixed with rare blasts showing prominent bright pink cytoplasmic granules and scant Auer rods.

**Figure 2:**
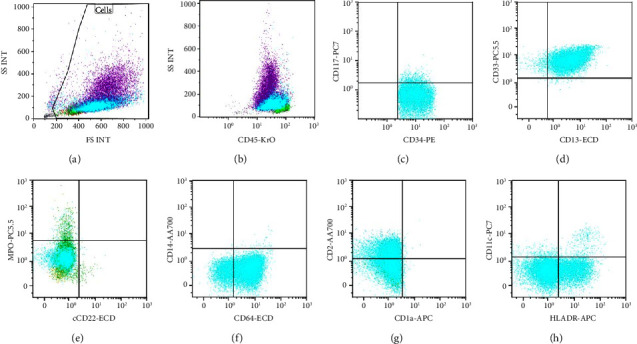
Blast population (aqua) gated based on (a) forward scatter and side scatter with (b) dimmer CD45 in a 10-color flow cytometric evaluation. Pertinent scatter plots show gated blasts are (c) positive for CD34 and CD117 (dim to negative), (d) positive for CD13 and CD33, (e) negative for CD22 and cMPO (cytoplasmic myeloperoxidase), (f) positive for CD64 and negative for CD14, (g) dim to negative for CD2 and negative for CD1a, and (h) partially positive (subset) for HLA-DR and negative for CD11c.

The peripheral blood smear showed medium to large blasts with predominantly bilobed nuclei. The cytoplasmic granularity was variable with several hypogranular forms intermixed with few showing prominent bright pink cytoplasmic granules and Auer rods (Figure 1). A population of abnormal cells comprising 35.68% of the total events was identified in the blast gate in a 10-color flow cytometric evaluation. The immunophenotype of the blasts is summarized in Table 1 and select dot plots are shown in Figures 2(a)–2(h).

**Figure 3:**
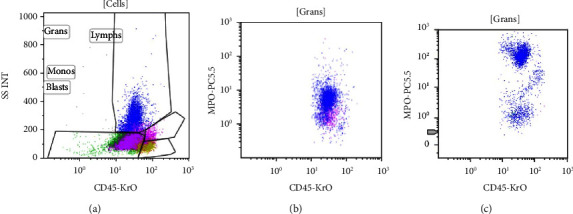
The residual normal granulocytes, from the patient sample, gated on side scatter CD45 plot (a) show moderate cMPO (myeloperoxidase). (b) The cMPO expression from a healthy control (c) is much brighter and is shown for comparison.

The blasts were negative for cMPO while residual normal granulocytes showed moderate cMPO as shown in Figures 3(a)–3(c). An underlying MPO deficiency was suspected for this atypical MPO expression pattern; however, the patient denied recurrent or frequent infections and her medical history was not consistent with MPO deficient phenotype.

**Figure 4:**
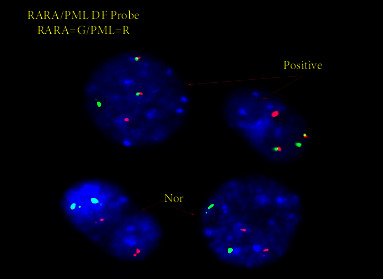
Fluorescence in situ hybridization analysis with the PML/RARA (15q24/17q21) dual color, dual fusion translocation DNA probe showing an abnormal hybridization signal pattern, that showed a fusion of the PML and RARA loci in interphase nuclei in a background of normal cells negative for the fusion.

Despite the MPO negativity, which is highly unusual, the immunophenotypic features were otherwise congruent with APL, especially considering the blast morphology which was highly suspicious for hypogranular APL. Fluorescence in situ hybridization analysis with the PML/RARA (15q24/17q21) dual color, dual fusion translocation DNA probe revealed an abnormal hybridization signal pattern, which showed a fusion of the PML and RARA loci in 95 of 100 (95%) interphase nuclei consistent with fusion of the PML/RARA due to the t(15;17)(q24.1; q21.2) anomaly, corroborating the diagnosis (Figure 4). Cytogenetic analysis of unstimulated peripheral blood cells revealed an abnormal female chromosome complement with a balanced translocation between the long arms of chromosomes 15 and 17 at bands (q24.1; q21.2) in 20 of 20 cells. A cell-based quantitative PCR for PML/RARA t(15;17) showed PML-RARA transcript ratio/normalized copy number of 807.008 using ABL1 as the internal control.

## 3. Discussion

APL, first reported by Hillestad et al. in 1957, was extensively characterized by Bernard et al, who described the promyelocytic proliferation, hyperacute presentation, and catastrophic coagulopathy [6]. The etiology of this acute myeloid leukemia variant is a differentiation block at the promyelocytic stage due to the dominant negative PML-RARA fusion protein, resulting from characteristic balanced chromosomal translocation between chromosomes 15 and 17 t(15;17) (q24; q21) is seen in 95% of cases [7, 8]. APL constitutes 10–15% of all acute myeloid neoplasms [9]. The discovery and subsequent therapeutic usage of differentiating agents all-trans retinoic acid (ATRA) and arsenic trioxide (ATO) in APL has led to the current remarkable cure rates.

A significant reduction in early death rates in APL was achieved by instituting ATRA treatment based on preliminary cytomorphologic and immunophenotypic diagnosis, which can be reported within a few hours without waiting for the genetic confirmation of the PML-RARA fusion [3]. Thus, the success rate of ATRA relies heavily on the early cytomorphologic and immunophenotypic diagnosis of APL. Variant morphologic presentations of APL, especially the microgranular variant, present a challenge and immunophenotyping becomes paradigm for diagnosis in these cases [5].

The predominant immunophenotypic pattern is seen in APL cases, CD34 and HLA-DR negative blasts, that are positive for CD13, CD33, and CD117 with a bright MPO. Microgranular APL and hypergranular APL are immunophenotypically similar, except for CD34 and CD2, which are more frequently positive in the microgranular variant [10]. Though several variations may be seen in the APL immunophenotype, MPO positivity is invariable to the point that, when a case of acute leukemia is negative for MPO, the diagnosis of APL is less likely [10]. Thus, MPO positivity is used as a strong adjunct to morphology in the preliminary diagnosis of APL, especially in the microgranular variant. MPO negativity in this morphological setting raises the possibility of monocytic leukemias [11].

The substantial reliability on MPO expression comes with the caveat of inherent MPO deficiency, which is the commonest inherited defect of phagocytes. MPO localizes to the azurophillic granules in the neutrophils and plays a key role in oxidative killing [12, 13]. Though MPO deficiency theoretically leads to impaired antimicrobicidal activity; however, majority of the MPO deficiencies are clinically asymptomatic and patients do not typically grapple with severe infections unless they have concurrent diabetes, in which case, they may suffer chronic, recurrent candida infections [12, 13]. Heterozygosity for MPO deficiency has almost zero clinical impact or implications. However, this inherited defect in MPO expression can result in altered immunophenotypic patterns in acute myeloid leukemia and are an important diagnostic pitfall.

Till date, only five cases of APL with reduced (2 cases) or absent MPO (3 cases) have been described in literature [4, 11, 14]. Of these, three were microgranular variants and two were classic APLs. Heiblig et al. extensively analyzed one of these cases of MPO negative microgranular APL and found germline heterozygous MPO loss of function mutation (*MPO* c.2031 -2A > C), with somatic uniparental disomy of 17q resulting in homozygous MPO deficiency and aberrant MPO negativity in the myeloblasts [11].

MPO deficiency was previously thought to be extremely rare [12]; however, recent studies have suggested that the incidence might be much higher, with a reported frequency of 1 per 200 to 4,000 people in the United states and Europe and 1 per 55,000 people in Japan [11–13]. The prevalence of heterozygotes for *MPO* c.2031-2A > C alone, which is one of several possib**l**e losses of function mutations of the *MPO* gene, is close to 1% in the population according to one study [15]. The identification of MPO deficiency based on genetic testing would involve at the least a MPO gene scanning with NGS, followed by microarray to identify somatic uniparental disomy in heterozygotes [11]. This testing is not only redundant given the scant clinical implications of heterozygous MPO deficiency but also time consuming.

An easy way to identify background MPO deficiency confounding the immunophenotype of a myeloid neoplasm is the MPO expression in background neutrophils gated on the initial flow cytometry. Lanza et al. [16] described three distinct patterns of MPO expression in the setting of MPO deficient phenotypes. Dim MPO was characteristic in hereditary total MPO deficiency, while bright MPO was typical of patients with secondary MPO deficiency. Subjects with heterozygous germline MPO deficiency showed a distinct medium MPO antigenic expression, as seen in the case highlighted in this report. The MPO expression in the background neutrophils in MPO deficient APL has not been explored in any of the 5 prior reports of this entity. A dim MPO in the background neutrophils, in the morphological setting of APL, can identify underlying MPO deficiency, clarifying immunophenotypic ambiguity and thus establishing an unequivocal diagnosis.

## Figures and Tables

**Table 1:** Immunophenotype of blasts.

Marker		Expression on cells (blasts) in the blast gate
Positive	CD2	Dim to negative
	CD9	Positive
	CD13	Positive
	CD33	Positive
	CD34	Positive
	CD38	Dim to negative
	CD45	Positive
	CD58	Positive
	CD64	Positive
	CD117	Dim to negative
	CD123	Bright
	HLA_DR	Partial, subset

Negative	CD1a, CD3, cCD3, CD4, CD5, CD7, CD8, CD10, CD11b, CD11c, CD14, CD15, CD16, CD19, CD20, CD22, cCD22, CD25, CD42b, CD56, CD61, cCD79a, CD235a, kappa, lambda, TdT, cMPO, and cIgM	
